# Prevalence of Social Anxiety and Its Association With Low Self-Esteem Among Medical Students: A Cross-Sectional Study From Kerala, India

**DOI:** 10.7759/cureus.113182

**Published:** 2026-07-22

**Authors:** Riya Mercy Jacob, Jaico K Paulose

**Affiliations:** 1 Radiology, Pushpagiri Medical College, Thiruvalla, IND; 2 Psychiatry, Believers Church Medical College Hospital, Thiruvalla, IND

**Keywords:** college mental health, low self-esteem, medical education interest, medical students' mental health, social anxiety, youth mental health

## Abstract

Background: Social anxiety is common among medical students because of academic pressure and frequent evaluations. Low self-esteem may contribute to the development of social anxiety. This study assessed the prevalence of social anxiety and low self-esteem and examined their association among medical students.

Objectives: This study aimed to determine the prevalence of social anxiety and low self-esteem among medical students and to evaluate the association between social anxiety and low self-esteem, as well as their relationship with gender.

Methods: A cross-sectional analytical study was conducted among MBBS students at Believers Church Medical College Hospital, Thiruvalla, Kerala, India, between 2019 and 2021. All MBBS students from the 2016-2021 admission batches were invited to participate through a self-administered online questionnaire (Google Forms, Google LLC, California, USA). Of 600 eligible students, 507 responded (84.5%). Social anxiety was assessed using the Social Interaction Anxiety Scale (SIAS) and self-esteem using the Rosenberg Self-Esteem Scale (RSES). Social anxiety was defined as an SIAS score ≥35 and low self-esteem as an RSES score <20. Data were analyzed using IBM SPSS Statistics for Windows, version 25 (IBM Corp., Armonk, NY, USA). Chi-square tests, odds ratios (ORs), Pearson correlation, and multivariable logistic regression were performed.

Results: Social anxiety was identified in 201 (39.64%) participants, while low self-esteem was present in 134 (26.43%). Low self-esteem was significantly associated with social anxiety (χ² = 67.41, p < 0.001; OR =5 .57, 95% confidence interval (CI): 3.62-8.56). Female participants had significantly higher odds of low self-esteem (OR = 2.31, p < 0.001), whereas gender was not independently associated with social anxiety. SIAS and RSES scores showed a significant negative correlation (r = -0.595, p < 0.001). Low self-esteem remained an independent predictor of social anxiety after adjustment for gender (adjusted OR = 5.76, 95% CI: 3.71-8.93; p < 0.001). Most participants reported symptom onset between 15 and 19 years.

Conclusions: Social anxiety and low self-esteem are highly prevalent among medical students. Low self-esteem is independently associated with social anxiety, highlighting the importance of early screening and interventions aimed at improving psychological well-being in this population.

## Introduction

Social anxiety is a chronic psychiatric condition characterized by marked fear or anxiety in situations where individuals may be observed or evaluated by others. Common manifestations include fear of public speaking, interacting with unfamiliar people, participating in group discussions, eating or writing in public, avoidance of social situations, excessive self-consciousness, anticipatory anxiety, blushing, trembling, sweating, palpitations, and impaired academic, occupational, and social functioning [1,2]. It typically manifests during adolescence and can lead to significant impairment in academic, occupational, and social functioning [2]. The lifetime prevalence of social anxiety in the general population has been estimated to range from 3% to 13%, whereas studies from India have reported substantially lower prevalence estimates in the general population. Community-based studies from India have reported prevalence estimates of approximately 2-5%, considerably lower than estimates reported in Western countries [3].

Contemporary neuropsychological models suggest that social anxiety arises from interactions between cognitive biases, altered emotional processing, self-efficacy, personality characteristics, and neural circuits involved in threat detection and emotion regulation. Individuals with social anxiety often demonstrate heightened sensitivity to perceived social threats, exaggerated amygdala responses to negative social cues, reduced confidence in their social abilities (self-efficacy), and greater levels of neuroticism and insecure personality traits. Social anxiety also shows considerable comorbidity with depression, generalized anxiety disorder, substance use disorders, and avoidant personality disorder, suggesting overlapping psychological and neurobiological mechanisms [2,4,5-8].

However, specific populations such as medical students are at increased risk due to academic stress, competitive environments, and frequent evaluation [9]. Among medical students, the prevalence is considerably higher than in the general population. A recent systematic review reported that approximately one-third of medical students worldwide experience clinically significant social anxiety symptoms, with prevalence varying according to screening instrument, diagnostic threshold, and geographical region [9]. Medical education exposes students to multiple psychological stressors, including a demanding curriculum, frequent written and practical examinations, bedside clinical assessments, oral viva examinations, competition for academic achievement, transition to patient care responsibilities, fear of making mistakes, and continuous evaluation by faculty and peers. These stressors may increase vulnerability to anxiety disorders, particularly social anxiety [9,10,11].

Self-esteem refers to an individual’s overall evaluation of personal worth and has an important role in emotional regulation, resilience, coping behavior, and psychological well-being [10]. Self-esteem plays a vital role in emotional regulation and resilience. According to the self-presentational model proposed by Schlenker and Leary, individuals with low self-esteem often perceive themselves as socially inadequate and doubt their ability to create favorable impressions on others [4]. These negative self-beliefs increase fear of criticism and rejection, resulting in heightened self-focused attention during social interactions. This excessive self-monitoring promotes anticipatory anxiety, avoidance of feared situations, and maintenance of social anxiety symptoms through negative reinforcement. Consequently, low self-esteem may both predispose individuals to social anxiety and perpetuate existing symptoms [4]. Individuals with healthy self-esteem generally demonstrate greater confidence, adaptive coping strategies, and resilience when confronted with stressful situations. Conversely, low self-esteem has consistently been associated with depression, anxiety disorders, impaired interpersonal functioning, reduced academic confidence, and poorer quality of life [4,10]. Self-efficacy and personality security may further influence this relationship by affecting confidence during social interactions and the ability to regulate emotional responses [4,7,8].

Despite its importance, limited studies have explored the relationship between self-esteem and social anxiety disorder among Indian medical students. Understanding this relationship is important for developing targeted interventions. This study aims to assess the prevalence of social anxiety disorder and low self-esteem among medical students and to evaluate their association with gender and age of onset.

## Materials and methods

Study design and setting

This cross-sectional analytical study was conducted among undergraduate medical (MBBS) students at Believers Church Medical College Hospital, Thiruvalla, Kerala, India, between January 2019 and December 2021.

Study population and sampling

The study population consisted of all MBBS students enrolled in the institution during the study period, including students from the 2016, 2017, 2018, 2019, 2020, and 2021 admission batches (first-year to final-year MBBS students).

Sample Size

A census sampling approach was used. All undergraduate MBBS students enrolled at Believers Church Medical College Hospital during the study period (2019-2021) were considered eligible for participation. Therefore, no formal sample size calculation was performed. Instead, all 600 eligible students from the 2016-2021 admission batches were invited to participate in order to maximize study power and minimize sampling bias. A total of 507 students completed the questionnaire, yielding a response rate of 84.5%. Although the response rate was high (84.5%), characteristics of non-responders were not available for comparison; therefore, the possibility of non-response bias cannot be completely excluded.

Inclusion and exclusion criteria

The study included all MBBS students enrolled at Believers Church Medical College Hospital, Thiruvalla, Kerala, India, during the study period (2019-2021) who provided informed consent to participate. Students who did not provide informed consent or submitted incomplete questionnaire responses were excluded from the study.

Data collection procedure

Data were collected over a three-year period (January 2019 to December 2021) using a structured, self-administered questionnaire distributed electronically through Google Forms (Google LLC, California, USA). Participation was voluntary, and responses were anonymous and confidential. The questionnaire consisted of demographic information, the Social Interaction Anxiety Scale (SIAS) [11,12,13], and the Rosenberg Self-Esteem Scale (RSES) [8]. Participants were also asked to report the age at which they first experienced symptoms related to social anxiety and low self-esteem.

Study instruments

The SIAS, developed by Mattick and Clarke, is a validated 20-item self-report instrument designed to assess distress associated with social interactions and fear of negative evaluation (see Appendix A) [11]. The SIAS has demonstrated excellent internal consistency, high test-retest reliability, and good convergent and discriminant validity [11]. Each item is rated on a five-point Likert scale with response options ranging from 0 = "not at all characteristic or true of me," 1 = "slightly characteristic or true of me," 2 = "moderately characteristic or true of me," 3 = "very characteristic or true of me," and 4 = "extremely characteristic or true of me." Items 5, 9, and 11 are reverse scored. Total scores range from 0 to 80, with scores ≥35 considered indicative of clinically significant social anxiety [11,13].

The RSES is a widely used and validated 10-item measure of global self-esteem with good reliability and construct validity across diverse populations [5,8,9]. Each item is rated on a four-point Likert scale with response options 0 = "strongly disagree," 1 = "disagree," 2 = "agree," and 3 = "strongly agree." Negatively worded items (2, 5, 6, 8, and 9) are reverse scored. Total scores range from 0 to 30 in the original scoring system; in the present study, responses were coded on a 0-4 scale (maximum score 40), with scores <20 considered indicative of low self-esteem (see Appendix B).

Both the SIAS and RSES were used solely for non-commercial academic research. To the best of our knowledge, no additional permission or licensing was required for this use. Therefore, no separate permission documents were obtained.

Outcome measures

The primary outcome measures were the prevalence of social anxiety and the prevalence of low self-esteem among medical students. Secondary outcome measures included the association between social anxiety and low self-esteem, the association of gender with social anxiety and low self-esteem, and the distribution of the reported age of onset of social anxiety-related difficulties.

Statistical analysis

Data were entered and analyzed using IBM SPSS Statistics for Windows, version 25 (IBM Corp., Armonk, NY, USA). Descriptive statistics were used to summarize participant characteristics and prevalence estimates. Associations between categorical variables were assessed using a chi-square (χ2) test. Odds ratios (ORs) with 95% confidence intervals (CIs) were calculated. Pearson and Spearman correlation analyses were performed to assess the relationship between SIAS and RSES scores. Multivariable logistic regression analysis was conducted with social anxiety as the dependent variable and relevant predictors entered into the model. Internal consistency of the SIAS and RSES was assessed using Cronbach's alpha. A p-value <0.05 was considered statistically significant. Because the primary analyses consisted of descriptive statistics, chi-square tests, odds ratios, correlation analyses, and logistic regression, formal assessment of homogeneity of variances was not applicable. Pearson and Spearman correlation coefficients were used to account for the distributional characteristics of the data.

Ethical considerations

Informed consent was obtained from all participants prior to inclusion. Participant anonymity and confidentiality were maintained throughout the study. The study was approved by the Institutional Ethics Committee at Believers Church Medical College, Thiruvalla, Kerala, India (Reg No.: ECR/1118/lNST/KL/2019). Before accessing the questionnaire, participants were presented with an electronic Participant Information Sheet describing the study objectives, voluntary nature of participation, expected time required, confidentiality measures, and contact details of the principal investigator. Participants were required to provide electronic informed consent by selecting an "I agree to participate" option before proceeding to the questionnaire. Those who declined consent could not access the survey. Responses were collected anonymously, and no personally identifying information was obtained. Consequently, individual participants with elevated SIAS or low RSES scores could not be identified or contacted. Participants were informed that the questionnaires were screening tools rather than diagnostic instruments and were advised to seek professional mental health evaluation if they experienced significant psychological distress.

## Results

A total of 507 medical students participated out of 600 invited students, yielding a response rate of 84.5%. Females constituted 323 (63.7%) participants, while males constituted 184 (36.3%) (Table 1).

**Table 1:** Demographics

Variable	n (%)
Total participants	507
Female	323 (63.7)
Male	184 (36.3)

Social anxiety was identified in 201 students (39.64%), while 306 students (60.36%) did not meet the cutoff for social anxiety. Low self-esteem was present in 134 students (26.43%), while 373 students (73.57%) had normal self-esteem (Table 2).

**Table 2:** Prevalence of social anxiety and low self-esteem

Variable	Number of participants (n)	Prevalence
Social anxiety	201	39.64%
Low self-esteem	134	26.43%

A statistically significant association was observed between low self-esteem and social anxiety. Among students with low self-esteem, 93 of 134 (69.40%) had social anxiety, compared with 108 of 373 (28.95%) students with normal self-esteem (Table 3). Students with low self-esteem had 5.57 times higher odds of social anxiety (OR = 5.57, 95% Cl: 3.62-8.56; χ2 = 67.41, p < 0.001).

**Table 3:** Association between social anxiety and low self-esteem OR = 5.57, 95% Cl: 3.62-8.56; χ2 = 67.41, p < 0.001

Self-esteem status	Social anxiety	No social anxiety
Low self-esteem	93	41
Normal self-esteem	108	265

Gender-stratified analysis showed that social anxiety was present in 131 of 323 females (40.56%) and 70 of 184 males (38.04%). This difference was not statistically significant (χ2= 0.31, p = 0.578; OR = 1.11, 95% Cl: 0.77-1.61) (Table 4).

**Table 4:** Gender- stratified distibution of social anxiety χ² = 0.31, p = 0.578; OR = 1.11, 95% CI: 0.77–1.61.

Gender	Social anxiety	No social anxiety
Female	131	192
Male	70	114

Low self-esteem was significantly more common among females, occurring in 103 of 323 females (31.89%), compared with 31 of 184 males (16.85%). Female students had 2.31 times higher odds of low self-esteem than male students (OR = 2.31, 95% CI: 1.47-3.63; χ2 = 13.64, p < 0.001) (Table 5). 

**Table 5:** Gender- stratified distibution of low self esteem χ² = 13.64, p < 0.001; OR = 2.31, 95% CI: 1.47–3.63.

Gender	Low self-esteem	Normal self-esteem
Female	103	220
Male	31	153

The most common reported age of onset of social anxiety-related symptoms was 15-19 years, reported by 259 students (51.08%), followed by 10-14 years in 105 students (20.71%) (Table 6). Correlation analysis showed a significant negative relationship between SIAS and RSES scores.

**Table 6:** Self-reported age of onset of symptoms of social anxiety or low self-esteem

Age group (years)	n (%)
5-9	29 (5.72%)
10-14	105 (20.71%)
15-19	259 (51.08%)
20-24	62 (12.23%)
25 or older	4 (0.79%)
Missing/not applicable	48 (9.47%)

The mean total SIAS score was 30.77 ± 15.58, with a median of 30, an interquartile range of 19-42, and an observed range of 0-77. The mean total RSES score was 24.54 ± 8.26, with a median of 24, an interquartile range of 19-31, and an observed range of 0-40. Both score distributions deviated significantly from normality on the Shapiro-Wilk test (p < 0.001) (Table 7).

**Table 7:** Descriptive statistics for the Social Interaction Anxiety Scale (SIAS) and self-esteem using the Rosenberg Self-Esteem Scale (RSES) scores

Instrument	Mean ± SD	Median (IQR)	Observed range
Social Interaction Anxiety Sale	30.77 ± 15.58	30 (19-42)	0-77
Rosenberg Self-Esteem Scale	24.54 ± 8.26	24 (19-31)	0-40

Pearson correlation was r = -0.595 (p < 0.001), and Spearman correlation was ρ = -0.576 (p < 0.001), indicating that lower self-esteem scores were associated with higher social anxiety scores (Table 8).

**Table 8:** Correlation between Social Interaction Anxiety Scale (SIAS) and Rosenberg Self-Esteem Scale (RSES) scores

Analysis	Correlation coefficient	p value
Pearson correlation	-0.595	<0.001
Spearman correlation	-0.576	<0.001

Multivariable logistic regression showed that low self-esteem remained independently associated with social anxiety after adjusting for gender (adjusted OR = 5.76, 95% CI: 3.71-8.93, p < 0.001) (Table 9). Female gender was not independently associated with social anxiety (adjusted OR = 0.84, 95% Cl: 0.56-1.25, p = 0.389).

**Table 9:** Multivariable logistic regression for predictors of social anxiety

Predictor	β coefficient	Standard error	Adjusted odds ratio	95% confidence interval	p value
Low self-esteem	1.751	0.224	5.76	3.17-8.93	<0.001
Female gender	-0.177	0.206	0.84	0.56-1.25	0.389

Internal consistency was excellent for both instruments, with Cronbach's alpha of 0.923 for the SIAS and 0.870 for the RSES (Table 10).

**Table 10:** Internal consistency of the Social Interaction Anxiety Scale (SIAS) and Rosenberg Self-Esteem Scale (RSES)

Instrument	Cronbach's alpha
SIAS	0.923
RSES	0.87

## Discussion

The present study demonstrated a high prevalence of social anxiety among medical students, with 201 of 507 participants (39.64%) meeting the predefined SIAS cutoff. The prevalence of social anxiety identified in the present study, 201 (39.64%), was substantially higher than the 3.6% current prevalence of anxiety disorders reported in the adult Indian general population by the National Mental Health Survey of India [3]. However, this comparison should be interpreted cautiously because the national estimate represented anxiety disorders collectively, whereas the present study specifically assessed social anxiety symptoms using the SIAS. The prevalence found in this study is consistent with previous studies suggesting that medical students represent a psychologically vulnerable group because of sustained academic demands, frequent assessments, and highly competitive learning environments [3,9]. This prevalence was substantially higher than the 7.8% reported by Honnekeri et al. among urban Indian undergraduate students using the SIAS and Social Phobia Scale [10] and the 9.6% prevalence reported by İzgiç et al. among Turkish university students [6]. However, it was lower than the 62.5% prevalence reported by Obadeji and Kumolalo among Nigerian undergraduate students [7]. These comparisons indicate that estimates of social anxiety among university students vary considerably across settings. Differences in the instruments used, cutoff scores, participant characteristics, academic disciplines, cultural attitudes toward social interaction, and timing of data collection may account for this variation [5-7]. However, direct comparisons should be interpreted cautiously because prevalence estimates vary according to study population, cultural context, screening instrument, cutoff score, and whether symptoms are identified through self-report screening or diagnostic interviews. The high prevalence observed in the present study is consistent with the broader evidence that students in demanding academic programmes experience a substantial burden of anxiety and psychological distress [9].

Several features of medical training may help explain the relatively high prevalence identified in this study. Medical students are repeatedly exposed to written examinations, oral viva examinations, bedside assessments, clinical case presentations, interactions with unfamiliar patients, and direct evaluation by faculty members and peers. These situations closely resemble the scrutiny-based and interpersonal circumstances that commonly provoke social anxiety. Sustained academic workload, competition, fear of committing errors, concern about professional competence, and limited time for rest may further increase vulnerability to psychological distress [4]. Anonymous self-administered screening may also identify students with significant symptoms who have not sought clinical help. Consequently, the present prevalence estimate represents screening-positive social anxiety and should not be interpreted as the prevalence of a formally diagnosed psychiatric disorder.

Low self-esteem was identified in 134 participants (26.43%). This estimate was comparable to the 24.1% prevalence reported by Alghamdi et al. among 1,099 medical students in Saudi Arabia [13]. The principal finding of this study was the strong association between low self-esteem and social anxiety. Participants with low self-esteem had more than fivefold higher odds of social anxiety in the unadjusted analysis (OR = 5.57), and this association remained statistically significant after adjustment for gender (adjusted OR = 5.76, 95% CI: 3.71-8.93). Obadeji and Kumolalo similarly reported that social anxiety scores were negatively correlated with self-esteem among undergraduate students [7]. İzgiç et al. also found significantly lower self-esteem among university students with social phobia than among those without social phobia [6]. In a college sample, Abdollahi and Talib reported that lower self-esteem was associated with greater social anxiety [8]. These studies support the present finding that impaired self-esteem is closely related to social anxiety across different student populations.

The continuous-score analysis further supported this association. Total SIAS and RSES scores showed a moderate-to-strong negative relationship in the present sample (Pearson’s r = −0.595 and Spearman’s ρ = −0.576; both p < 0.001). The magnitude of this relationship was stronger than the correlation reported by Ayed et al. among nursing students [12]. Differences in participant characteristics, scoring methods, symptom severity, academic setting, and sociocultural background may partly explain the variation in effect size. Importantly, the continuous relationship indicates that the association is not limited to students who cross a categorical cutoff; progressively lower self-esteem was associated with progressively higher social anxiety scores.

The relationship between the two constructs can be understood through cognitive and self-presentational models of social anxiety. Schlenker and Leary proposed that social anxiety develops when individuals are motivated to make a favourable impression but doubt their ability to do so successfully [5]. Students with low self-esteem may interpret themselves as socially inadequate, underestimate their communication ability, anticipate rejection or criticism, and monitor their behaviour excessively during interactions. This heightened self-focused attention can increase anticipatory anxiety and encourage avoidance. Although avoidance may produce short-term relief, it prevents corrective social experiences and may reinforce both negative self-beliefs and anxiety over time [5]. Therefore, low self-esteem may act as both a vulnerability factor and a maintaining correlate of social anxiety; however, the cross-sectional design of this study prevents determination of the temporal or causal direction.

Female participants had significantly higher odds of low self-esteem than male participants (OR = 2.31, 95% CI: 1.47-3.63), although gender was not independently associated with social anxiety. This finding is consistent with Alghamdi et al., who identified female gender as an independent predictor of low self-esteem among medical students [12]. Previous research has also shown that female medical students may report lower confidence and greater anxiety regarding competence despite academic performance comparable to that of male students [14,15,16]. Possible explanations include differences in self-appraisal, interpersonal expectations, perceived performance pressure, and sensitivity to external evaluation. Nevertheless, because socioeconomic status, age, academic year, academic performance, psychiatric history, and other potential confounders were not collected in the present study, the observed gender association should be interpreted cautiously.

Most participants reported onset of social anxiety- or low-self-esteem-related difficulties between 15 and 19 years of age. This finding is consistent with epidemiological evidence that social anxiety commonly begins during adolescence and early adulthood [2,15,9]. Adolescence involves major changes in identity, peer relationships, educational demands, and sensitivity to social evaluation. Early identification is particularly relevant in medical education because persistent social anxiety may interfere with classroom participation, oral examinations, communication training, clinical interaction, teamwork, and professional confidence.

The present study has several strengths. It included a relatively large sample of medical students from multiple MBBS admission batches, achieved an 84.5% response rate, and used established psychometric instruments. The relationship between social anxiety and self-esteem was examined using complementary categorical, correlational, and multivariable methods. The Social Interaction Anxiety Scale and Rosenberg Self-Esteem Scale also demonstrated excellent internal consistency in the present sample, with Cronbach’s alpha values of 0.923 and 0.870, respectively.

The study also has important limitations. Its cross-sectional design precludes causal inference. Questionnaire responses were self-reported and may therefore have been affected by recall, response, and social-desirability bias. The instruments were used for screening and cannot substitute for structured clinical assessment. The study was conducted at a single institution, limiting generalizability. Variables such as current age, socioeconomic status, academic year, academic performance, psychiatric history, place of residence, and social support were not collected, which restricted adjustment for potential confounding. Furthermore, the characteristics of the 93 non-respondents were unavailable, and non-response bias cannot be excluded. Future multicentre longitudinal studies incorporating broader demographic, academic, and clinical variables are needed to clarify the direction of the relationship and evaluate interventions aimed at strengthening self-esteem and reducing social anxiety.

## Conclusions

Social anxiety was identified in nearly two-fifths of the medical students in this study. Low self-esteem was strongly and independently associated with social anxiety, and lower self-esteem scores were correlated with greater social anxiety severity. Female participants had higher odds of low self-esteem, although gender was not independently associated with social anxiety. These findings identify self-esteem as an important psychological correlate of social anxiety among medical students. Early screening, accessible counselling, and interventions aimed at strengthening self-esteem, resilience, communication confidence, and psychological well-being may help support vulnerable students. Multicentre longitudinal research is required to clarify the temporal relationship between self-esteem and social anxiety and to evaluate the effectiveness of targeted interventions.

## Appendices

Appendix A: Social Interaction Anxiety Scale (SIAS)

Gender

Male

Female

Instructions

For each question, please fill in the blank with a number to indicate the degree to which you feel the statement is characteristic or true of you. The rating scale is as follows: 

0 = Not at all characteristic or true of me

1 = Slightly characteristic or true of me

2 = Moderately characteristic or true of me

3 = Very characteristic or true of me

4 = Extremely characteristic or true of me

1. I get nervous if I have to speak with someone in authority (e.g., a teacher).

2. I have difficulty making eye contact with others.

3. I become tense if I have to talk about myself or my feelings.

4. I find it difficult mixing comfortably with the people I work with.

5. *I find it easy to make friends of my own age.

6. I tense up if I meet an acquaintance in the street.

7. When mixing socially, I am uncomfortable.

8. I feel tense if I am alone with just one person.

9. *I am at ease meeting people at parties, etc.

10. I have difficulty talking with other people.

11. *I find it easy to think of things to talk about.

12. I worry about expressing myself in case I appear awkward.

13. I find it difficult to disagree with another’s point of view.

14. I have difficulty talking to attractive persons of the opposite sex.

15. I find myself worrying that I won’t know what to say in social situations.

16. I am nervous mixing with people I don’t know well. 

17. I feel I’ll say something embarrassing when talking.

18. When mixing in a group, I find myself worrying I will be ignored.

19. I am tense mixing in a group.

20. I am unsure whether to greet someone I know only slightly.

Appendix B: Rosenberg Self-Esteem Scale (RSES)

Instructions

Below is a list of statements dealing with your general feelings about yourself. Each item is to be rated on a four-point Likert scale: strongly agree (SA = 3), agree (A = 2), disagree (D = 1), and strongly disagree (SD = 0).

1. On the whole, I am satisfied with myself.

2.* At times, I think I am no good at all.

3. I feel that I have a number of good qualities.

4. I am able to do things as well as most other people.

5.* I feel I do not have much to be proud of.

6.* I certainly feel useless at times.

7. I feel that I’m a person of worth, at least on an equal plane with others.

8.* I wish I could have more respect for myself.

9.* All in all, I am inclined to feel that I am a failure. 10. I take a positive attitude toward myself.

At what age did most of the difficulties arise, if they did arise? 

A. 5-9 years

B. 10-14 years

C. 15-19 years

D. 20-24 years

E. 25 years or more
